# Influence of skeletal muscle volume loss during lenvatinib treatment on prognosis in unresectable hepatocellular carcinoma: a multicenter study in Tohoku, Japan

**DOI:** 10.1038/s41598-022-10514-3

**Published:** 2022-04-20

**Authors:** Masashi Fujita, Kazumichi Abe, Hidekatsu Kuroda, Takayoshi Oikawa, Masashi Ninomiya, Atsushi Masamune, Kazuo Okumoto, Tomohiro Katsumi, Wataru Sato, Katsunori Iijima, Tetsu Endo, Shinsaku Fukuda, Nobukazu Tanabe, Hiroshi Numao, Yasuhiro Takikawa, Yoshiyuki Ueno, Hiromasa Ohira

**Affiliations:** 1 Department of Gastroenterology, Fukushima Medical University School of Medicine, 1 Hikarigaoka, Fukushima City, Fukushima, 960-1295 Japan; 2 Division of Hepatology, Department of Internal Medicine, Iwate Medical University, Morioka, Japan; 3 Division of Gastroenterology, Tohoku University Graduate School of Medicine, Sendai, Japan; 4 Department of Gastroenterology, Faculty of Medicine, Yamagata University, Yamagata, Japan; 5 Department of Gastroenterology, Akita University School of Medicine, Akita, Japan; 6 Department of Gastroenterology, Hirosaki University School of Medicine, Hirosaki, Japan; 7 Department of Gastroenterology, National Hospital Organization Sendai Medical Center, Sendai, Japan; 8 Department of Gastroenterology, Aomori Prefectural Central Hospital, Aomori, Japan

**Keywords:** Cancer, Diseases, Risk factors

## Abstract

Sarcopenia is associated with poor prognosis of patients with hepatocellular carcinoma (HCC). We investigated the association of skeletal muscle volume (SMV) and its change in HCC patients taking lenvatinib. In 130 HCC patients, psoas mass index (PMI) was calculated as the left–right sum of the major × minor axis of psoas muscle at the third lumbar vertebra, divided by height squared. Patients were classified into two groups (low and normal PMI) based on indices of < 6.0 cm^2^/m^2^ for man and < 3.4 cm^2^/m^2^ for women. Change in PMI per month during the lenvatinib administration period (ΔPMI/m) was calculated; and patients were classified into two groups (severe and mild atrophy) based on the ΔPMI/m rate, as ≥ 1% or < 1%, respectively. There was no significant difference in Overall survival (OS) between the low and normal PMI groups at the start of lenvatinib administration. OS was significantly lower in the severe atrophy group than in the mild atrophy group (median; 15.2 vs. 25.6 months, *P* = 0.005). Multivariate analysis revealed a significant association of severe atrophy with OS (hazard ratio 1.927, *P* = 0.031). Progressive loss of SMV is a strong predictor of poor prognosis in HCC patients taking lenvatinib.

## Introduction

Hepatocellular carcinoma (HCC) is the sixth most common malignancy worldwide^1^. Until 2019, the clinical guidelines of the Japan Society of Hepatology (JSH)^2^. specified therapy with molecular targeting agents (MTAs), including sorafenib (SOR)^3^, regorafenib^4^, lenvatinib (LEN)^5^. and ramucirumab^6^, as the standard treatment for patients with unresectable HCC (u-HCC) and vascular invasion or extrahepatic metastasis. In 2020, immune checkpoint inhibitor (ICI) therapy including the combination of atezolizumab and bevacizumab^7^. was approved in Japan as the first-line treatment for unresectable HCC^8^. However, MTAs are still used as the second-line treatment and remain important because of the convenience of the administration method.

Skeletal muscle volume (SMV) loss is an important factor of sarcopenia that is associated with liver disease. Hanai et al. reported that SMV decreased by 2.2% per year in patients with liver cirrhosis, and the rate of decrease increased in parallel with the severity of cirrhosis^9^. Sarcopenia has a negative impact on mortality in patients with chronic liver disease (CLD)^10^. and u-HCC treated with SOR^11,12^. and LEN^13,14^. Several studies have reported that decline of SMV during the treatment period is a critical factor in patients with HCC treated with SOR or LEN^12,15^. However, these reports estimated change in SMV only over a short period from the start of LEN.

We have previously reported that change in the skeletal muscle index (SMI) was a risk factor in the patients with HCC patients treated with transarterial chemoembolization^16^. In that study, we used the psoas muscle index (PMI) as a simpler method for determining SMI, calculated as the vertical × horizontal diameter of the psoas muscle at the third lumbar vertebra (L3) on computed tomography (CT), divided by height squared^17^.

The present study aimed to evaluate the long-term effects of SMV loss in patients with u-HCC by examining changes in PMI at the start of LEN administration (PMI-Pre), at the time of the first judgement of therapeutic effect (PMI-1st), and at the end of LEN administration (PMI-Post).

## Results

### Baseline characteristics

Table 1 shows the baseline (Pre) characteristics of patients. Median age of the 130 patients was 70 years (range, 38–89 years) and 107 patients (82.3%) were men. The median observation period after the start of LEN administration was 11.0 months (range, 1.9–32.7 months). The median HCC treatment period before administration of LEN was 14.8 months (range, 0–132.8 months). The causative disease of HCC was hepatitis B virus (HBV; *n* = 28), hepatitis C virus (HCV; *n* = 35), alcohol (*n* = 37), non-alcoholic fatty liver disease (NAFLD; *n* = 26), and other (*n* = 4). Fourteen patients (10.8%) had moderate loss of liver function reserve (Child–Pugh class B). The median albumin-bilirubin (ALBI) score was − 2.38 and 51 patients (39.2%) had loss of liver function reserve (modified ALBI [mALBI] grade 2b or 3). Forty-nine patients (37.7%) had macrovascular invasion (MVI) and 36 patients (27.7%) had extrahepatic metastasis. Extrahepatic metastasis was to lymph nodes (*n* = 11), lung (*n* = 19, bone (*n* = 7), adrenal glands (*n* = 7), and carcinomatous peritonitis (*n* = 5). One-hundred-eleven patients (85.3%) had received other treatment prior to administration of LEN. The breakdown of treatment was as follows: surgery (*n* = 25), local treatment (*n* = 12), hepatic intra-arterial therapy (*n* = 70), radiation therapy (*n* = 2), and other treatment (*n* = 2). The median administration period was 7.7 months (range, 0.3–32.7 months). The initial dose of LEN was reduced in 37 patients and the dose was reduced during LEN treatment in 64 patients. Ninety-seven patients (74.6%) discontinued LEN, most commonly due to progressive disease (PD) (*n* = 44). Fifty-two patients (40.0%) died during the follow-up period.

**Table 1:** Baseline characteristics of participants.

Variable	Total (*n* = 130)
Observation period after initiation of LEN (months)	11.0 (7.7–17.2)
Sex (male/female)	107/23
Age (years)	70 (65–76)
Body weight (kg)	61.8 (55.2–69.0)
BMI (kg/m^2^)	23.5 (21.2–25.3)
Etiology (HBV/HCV/alcohol/NAFLD/other)	28/35/37/26/4
Child–Pugh class (A/B)	116/14
mALBI grade (1/2a/2b/3)	48/31/47/4
TNM stage (II/III/IVA/IVB)	21/47/26/36
Maximum tumor diameter (cm)	4 (2.5–7.5)
Number of tumors	4 (2–10)
Up-to-7 criteria (in/out/no liver tumor)	49/70/11
Macrovascular invasion (yes/no/no liver tumor)	49/70/11
Metastasis (yes/no)	36/94
HCC (recurrence/naive)	111/19
Total bilirubin (mg/dL)	0.8 (0.7–1.0)
Albumin (g/dL)	3.7 (3.4–4.1)
Prothrombin time-international normalized ratio	1.05 (0.97–1.13)
Platelet count (× 10^4^/μL)	14.7 (10.1–18.6)
Choline-esterase (U/L)**	198 (151–258)
Total cholesterol (mg/dL)**	165 (147–181)
Triglyceride (mg/dL)**	98 (75–131)
LDL-C (mg/dL)**	89 (69–115)
Hemoglobin A1c (%)**	5.9 (5.5–6.5)
Ammonia (μg/dL)**	48 (37–63)
AFP (ng/dL)	56.7 (7.3–669)
PIVKA-II (mAU/mL)	483.5 (55.5–2094)
Initial dose of LEN (4/8/12 mg)	8/76/46
Initial dose down (yes/no)	37/93
Dose down during administration (yes/no/unknown)	63/63/4
Administration period of LEN (months)	7.7 (3.1–12.2)
Discontinued LEN (yes/no)	97/33
Reason for discontinuing LEN (adverse event/PD/other)	39/44/14
1st mRECIST assessment (CR/PR/SD/PD/no assessment)	0/45/38/42/5
Received other treatments after discontinuing LEN (yes/no)	59/38
PMI (cm^2^/m^2^)	5.62 (4.63–6.87)
Male (cm^2^/m^2^)	5.73 (4.79–7.23)
Female (cm^2^/m^2^)	4.63 (3.55–5.64)
ΔPMI/m (cm^2^/m^2^)	0.05 (− 0.01 to 0.19)
ΔPMI/m rate (%)	0.99 (− 0.12 to 3.80)
Death (yes/no)	52/78

Values are presented as the median (interquartile range). *LEN* lenvatinib, *BMI* body mass index, *HBV* hepatitis B virus, *HCV* hepatitis C virus, *NAFLD* non-alcoholic fatty liver disease, *mALBI* modified albumin-bilirubin, *TNM* tumor node metastasis, *HCC* hepatocellular carcinoma, *LCL-C* low density lipoprotein cholesterol, *AFP* alpha fetoprotein, *PIVKA-II* protein induced by Vitamin K absence or antagonists-II, *PD* progressive disease, *mRECIST* Modified Response Evaluation Criteria in Solid Tumors, *CR* complete response, *PR* partial response, *SD* stable disease, *PMI* psoas muscle index, *ΔPMI/m* change in PMI per month, *ΔPMI/m rate* rate of change in PMI per month during administration of LEN.

**Calculated using the available data.

The median PMI-Pre by etiology was 5.84 cm^2^/m^2^ for HBV, 5.52 cm^2^/m^2^ for HCV, 6.39 cm^2^/m^2^ for alcohol, 5.32 cm^2^/m^2^ for NAFLD, and 5.16 cm^2^/m^2^ for other. The median change in PMI per month (ΔPMI/m) rate (ΔPMI*100/PMI-Pre) by etiology was 0.55% for HBV, 0.56% for HCV, 1.35% for alcohol, 2.67% for NAFLD, and 1.87% for other. There was no significant difference in PMI-Pre or ΔPMI/m rate in terms of etiology (*P* = 0.214 and *P* = 0.083, respectively).

### Change in PMI during the LEN administration period

**Figure 1:**
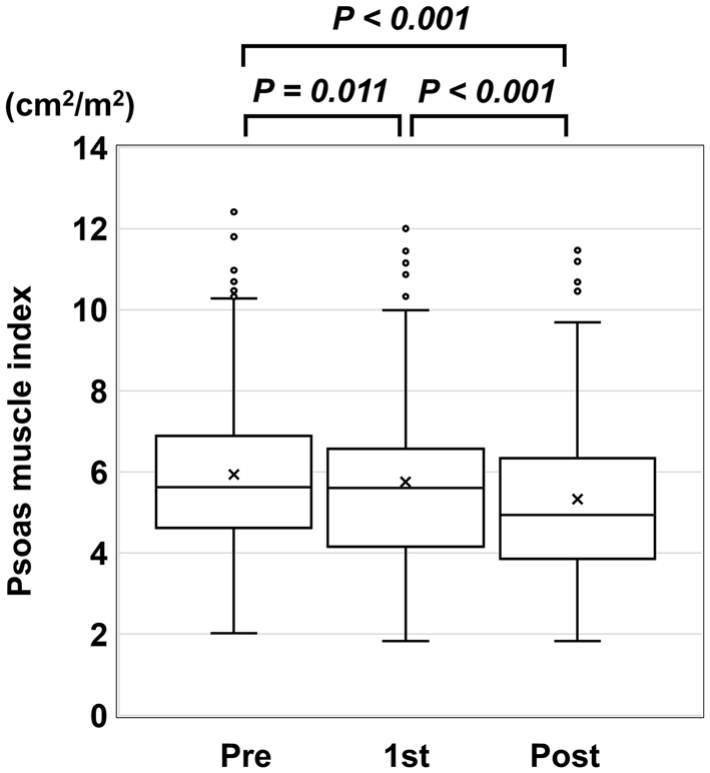
Transition of change in psoas muscle index (PMI) before (pre), during (1st), and after (post) administration of lenvatinib. There was a significant decrease in PMI at each successive measurement during the study period.

For all patients, median PMI-Pre, PMI-1st, and PMI-Post were 5.62, 5.61, and 4.94 cm^2^/m^2^, respectively. A significant difference was found between each of PMI-Pre and PMI-1st (*P* = 0.014), PMI-Pre and PMI-Post (*P* < 0.001), and PMI-1st and PMI-Post (*P* < 0.001) (Fig. 1). Median change in PMI per month during the LEN administration period (ΔPMI/m) was 0.05 cm^2^/m^2^ (interquartile range [IQR], − 0.01 to 0.19). And median rate of decrease of PMI per month (ΔPMI/m rate) was 0.99% (IQR, − 0.12 to 3.80), respectively (Table 1).

### Comparison between the low and normal PMI groups at baseline

Of the 130 patients, 63 (48.5%) were classified as having low PMI. There was no significant difference between the low and normal PMI groups in terms of age, liver function reserve (median ALBI score, − 2.42 vs. − 2.35; *P* = 0.889), stage of HCC including MVI and extrahepatic metastasis, or treatment (the presence, absence, or breakdown of other treatments received before LEN treatment). Significantly more men had low PMI than normal PMI (*P* = 0.005) (Table 2). Fewer patients in the low PMI group (PMI < 6.0 cm^2^/m^2^ for men and < 3.4 cm^2^/m^2^ for women) received other treatments after discontinuing LEN compared with those in the normal PMI group (PMI ≥ 6.0 cm^2^/m^2^ for men and ≥ 3.4 cm^2^/m^2^ for women) (*P* = 0.006). There was no significant difference in ΔPMI/m (*P* = 0.226) or in ΔPMI/m rate (*P* = 0.539) between the low and normal PMI groups.

**Table 2:** Comparison between the low and normal PMI groups at baseline.

Variable	Normal PMI (*n* = 67)	Low PMI (*n* = 63)	*P*-value
Observation period after initiation of LEN (months)	12.3 (8.4–17.3)	9.8 (7.3–17.1)	0.18
Sex (male/female)	**49/18**	**58/5**	**0.005**
Age (years)	70 (65–76)	70 (65–76)	0.725
Body weight (kg)	62.8 (56.0–68.7)	60.9 (54.5–69.2)	0.415
BMI (kg/m^2^)	23.9 (22.1–25.9)	23.0 (20.6–25.0)	0.087
Child–Pugh class (A/B)	61/6	55/8	0.496
mALBI grade (1/2a/2b/3)	26/16/24/1	22/15/23/3	0.532
TNM stage (I/II/III/IVA/IVB)	13/19/14/21	8/28/12/15	0.585
Up-to-7 criteria (in/out/no liver tumor)	23/36/8	26/34/3	0.457
Macrovascular invasion (yes/no/no liver tumor)	27/32/8	22/38/3	0.354
Metastasis (yes/no)	21/46	15/48	0.341
HCC (recurrence/naive)	57/10	54/9	0.921
Total bilirubin (mg/dL)	0.9 (0.7–1.0)	0.8 (0.7–1.0)	0.664
Albumin (g/dL)	3.7 (3.4–4.0)	3.7 (3.4–4.1)	0.83
Prothrombin time-international normalized ratio	1.05 (0.98–1.13)	1.04 (0.96–1.13)	0.432
Platelet count (× 10^4^/μL)	14.1 (9.9–17.0)	15.3 (12.1–20.7)	0.111
Choline-esterase (U/L)**	191 (155–239)	207 (136–271)	0.511
Total cholesterol (mg/dL)**	172 (157–186)	162 (143–180)	0.096
Triglyceride (mg/dL)**	104 (78–139)	93 (66–114)	0.093
LDL-C (mg/dL)**	91 (71–118)	89 (62–113)	0.279
Hemoglobin A1c (%)**	5.9 (5.4–6.6)	5.8 (5.5–6.5)	0.672
Ammonia (μg/dL)**	48 (37–63)	46 (39–63)	0.771
AFP (ng/dL)	97.2 (8.1–1875)	25.0 (6.7–354)	0.119
PIVKA-II (mAU/mL)	460 (37–2133)	485 (65–2047)	0.949
Initial dose of LEN (4/8/12 mg)	3/37/27	5/39/19	0.185
Initial dose down (yes/no)	16/51	21/42	0.236
Dose down during administration (yes/no/unknown)	34/31/2	29/32/2	0.596
Administration period of LEN (months)	8.2 (3.3–12.5)	6.7 (2.6–12.0)	0.267
Discontinued LEN (yes/no)	**45/22**	**52/11**	**0.045**
Reason for discontinuing LEN (adverse event/PD/other)	16/20/9	23/24/5	0.39
1st mRECIST assessment (CR/PR/SD/PD/no assessment)	**0/28/20/17/2**	**0/17/18/25/3**	**0.044**
Received other treatments after discontinuing LEN (yes/no)	**34/11**	**25/27**	**0.006**
ΔPMI/m (cm^2^/m^2^)	0.07 (0.00–0.26)	0.05 (–0.01–0.16)	0.226
ΔPMI/m rate (%)	1.13 (–0.02–4.04)	0.91 (–0.26–3.49)	0.539
Death (yes/no)	24/43	28/35	0.319

Significant values are in [bold].

Values are presented as the median (interquartile range). *LEN* lenvatinib, *BMI* body mass index, *mALBI* modified albumin-bilirubin, *TNM* tumor node metastasis, *HCC* hepatocellular carcinoma, *LCL-C* low density lipoprotein cholesterol, *AFP* alpha fetoprotein, *PIVKA-II* protein induced by Vitamin K absence or antagonists-II, *PD* progressive disease, *mRECIST* Modified Response Evaluation Criteria in Solid Tumors, *CR* complete response, *PR* partial response, *SD* stable disease, *PMI* psoas muscle index, *ΔPMI/m* change in PMI per month, *ΔPMI/m rate* rate of change in PMI per month during administration of LEN.

Low PMI group: PMI < 6.0 cm^2^/m^2^ for men and < 3.4 cm^2^/m^2^ for women, normal PMI group: PMI ≥ 6.0 cm^2^/m^2^ for men and ≥ 3.4 cm^2^/m^2^ for women.

**Calculated using the available data.

### Assessment of prognosis

**Figure 2:**
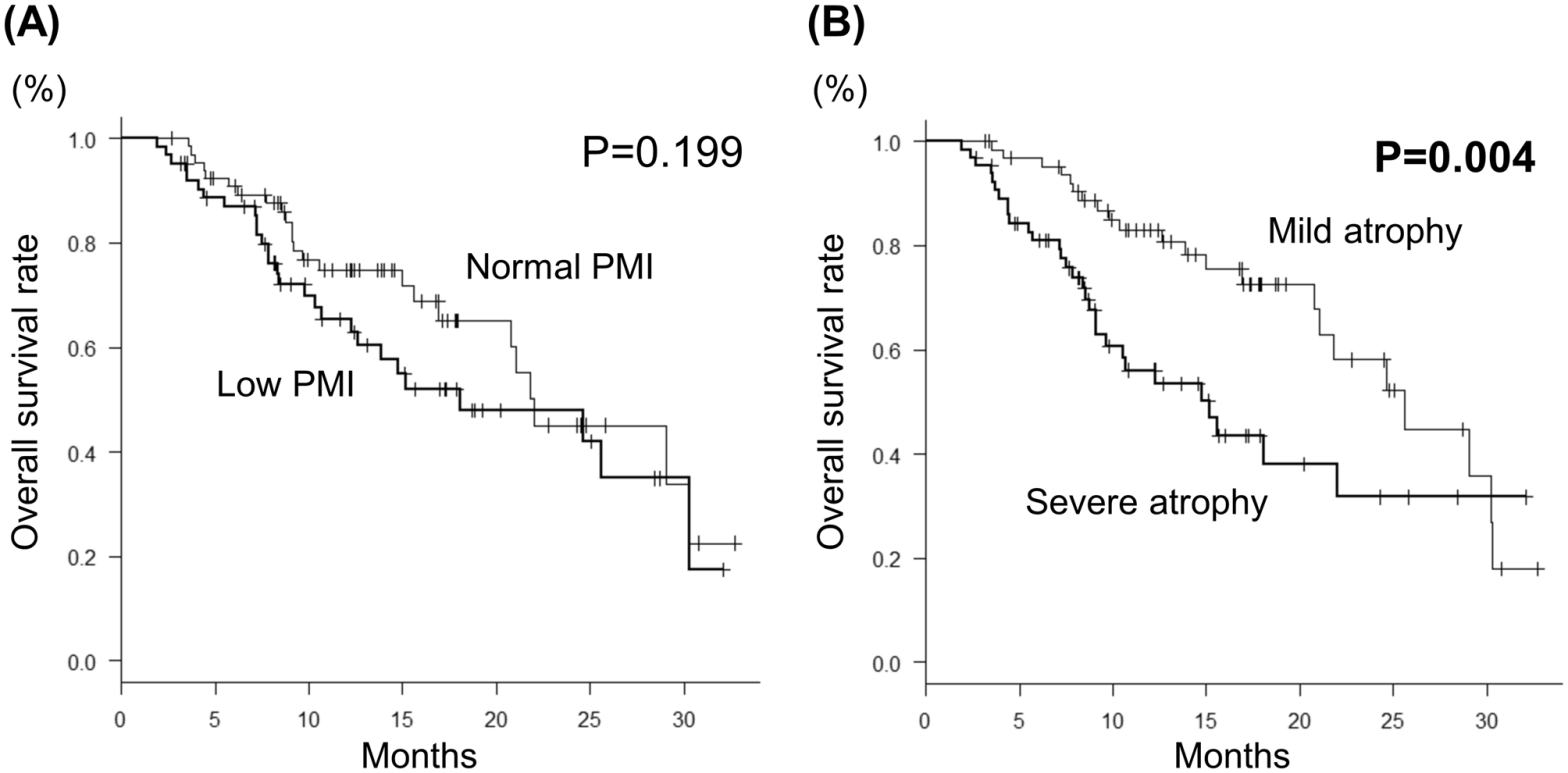
Kaplan–Meier curves for overall survival in the low and normal PMI groups (**A**) and in the severe and mild atrophy groups (**B**).

For all patients, median survival time (MST) was 21.8 months, and the survival rate at 12 and 24 months was 70.2% and 45.9%, respectively. There was no significant difference in MST between the low and normal PMI groups (18.1 month vs. 22.0 months, *P* = 0.199) (Fig. 2A).

Univariate analysis revealed the following as significantly associated with overall survival (OS, months): mALBI grade 2b or 3, tumor node metastasis (TNM) stage, alpha fetoprotein (AFP) ≥ 56.7 ng/dL, receiving other treatments after discontinuing LEN, and severe muscle atrophy (ΔPMI/m ≥ 1%) (Table 3). Multivariate analysis indicated the following as significantly associated with OS: severe muscle atrophy (hazard ratio [HR], 1.927; 95% confidence interval [CI], 1.061–3.500; *P* = 0.031), mALBI grade 2b or 3 (HR, 2.431; 95% CI, 1.333–4.434; *P* = 0.003), and AFP ≥ 56.7 ng/dL (HR, 2.563; 95% CI, 1.352–4.859; *P* = 0.004). Prognosis was significantly worse in the severe atrophy group (n = 65) than in the mild atrophy group (ΔPMI/m < 1%) (MST: 15.2 months vs. 25.6 months, *P* = 0.005) (Fig. 2B). Survival rates at 12 and 24 months in the severe and mild atrophy groups were 56.1%/31.8% and 83.0%/58.1%, respectively.

**Table 3:** Predictive factors for overall survival.

Variable	Univariate analysis		Multivariate analysis	
	HR (95% CI)	*P* value	HR (95% CI)	*P* value
Sex (male vs. female)	0.761 (0.367–1.577)	0.463	1.129 (0.507–2.512)	0.767
Age (years, ≥ 70 vs. < 70)	0.844 (0.487–1.462)	0.545	0.951 (0.532–1.702)	0.866
mALBI grade (2b/3 vs. 1/2a)	**2.994 (1.694–5.292)**	** < 0.001**	**2.431 (1.333–4.434)**	**0.003**
TNM stage (IVA/IVB vs. II/III)	**2.047 (1.167–3.592)**	**0.012**	1.676 (0.928–3.028)	0.087
AFP (ng/dL, ≥ 43 vs. < 43)	**3.185 (1.723–5.886)**	** < 0.001**	**2.563 (1.352–4.859)**	**0.004**
Received other treatments after discontinuing LEN (yes vs. no)	**0.414 (0.225–0.763)**	**0.005**	0.793 (0.419–1.501)	0.476
Low PMI vs. normal PMI	1.428 (0.827–2.467)	0.201		
Severe atrophy group vs. mild atrophy group	**2.173 (1.243–3.796)**	**0.006**	**1.927 (1.061–3.500)**	**0.031**

Significant values are in [bold].

*HR* hazard ratio, *CI* confidence interval, *mALBI* modified albumin-bilirubin, *TNM* tumor node metastasis, *AFP* alpha fetoprotein, *PIVKA-II* protein induced by Vitamin K absence or antagonists-II, *LEN* Lenvatinib, *PMI* psoas muscle index.

Low PMI group: PMI < 6.0 cm^2^/m^2^ for men and < 3.4 cm^2^/m^2^ for women, Normal PMI group: PMI ≥ 6.0 cm^2^/m^2^ for men and ≥ 3.4 cm^2^/m^2^ for women.

Severe atrophy group: ΔPMI/m rate ≥ 1%, Mild atrophy group: ΔPMI/m rate < 1%.

(ΔPMI/m rate: rate of change in psoas muscle index per month during administration of LEN).

For all patients, the median post progression survival (PPS) was 7.8 months, and the survival rate at 12 and 24 months was 35.5% and 24.8%, respectively. There was no significant difference in the median PPS between the low and normal PMI groups (7.6 month vs. 9.1 months, *P* = 0.481). On the other hand, PPS was significantly worse in the severe atrophy group than in the mild atrophy group (4.6 months vs. 11 months, *P* = 0.04).

### Characteristics of the severe and mild atrophy groups during the administration period

Table 4 shows the comparison of characteristics between the mild and severe atrophy groups during the administration period. The observation period was significantly shorter in the severe atrophy group than in the mild atrophy group (median; 8.7 months vs. 13.8 months, *P* < 0.001). The Pre values of choline-esterase (ChE; *P* = 0.024) were lower in the severe atrophy group than in the mild atrophy group. In contrast, the Post values of liver function reserve including Child–Pugh and ALBI score, albumin, and ChE were significantly worse in the severe atrophy group than in the mild atrophy group.

**Table 4:** Comparison between the severe and mild atrophy groups.

Variable	Time of start of taking LEN			Time of end of taking LEN		
	Mild atrophy (*n* = 65)	Severe atrophy (*n* = 65)	*P* value	Mild atrophy	Severe atrophy	*P* value
Observation period after initiation of LEN (months)	**13.8 (9.2–18.9)**	**8.7 (6.0–14.8)**	** < 0.001**			
Sex (male/female)	54/11	53/12	0.822			
Age (years)	70 (66–76)	70 (64–75)	0.597			
Body weight (kg)	62.8 (57.4–69.5)	60.7 (55.0–68.0)	0.174			
BMI (kg/m^2^)	23.9 (22.0–25.7)	22.6 (20.8–25.1)	0.054			
Child–Pugh score (5/6/7/8/9/10-/unknown)	42/18/4/1/0/0/0	37/19/6/2/1/0/0	0.285	**17/11/8/6/0/0/23**	**8/17/7/9/2/4/18**	**0.014**
Child–Pugh class (A/B/C/unknown)	60/5/0/0	56/9/0/0	0.261	28/14/0/23	25/18/4/18	0.126
ALBI score	–2.48 (–2.77 to –2.14)	–2.31 (–2.65 to –2.08)	0.129	**–2.28 (–2.51 to –1.95)**	**–2.05 (–2.39 to –1.59)**	**0.049**
mALBI grade (1/2a/2b/3/unknown)	29/13/22/1/0	19/18/25/3/0	0.114	8/10/19/5/23	5/8/26/8/18	0.137
TNM Stage (II/III/IVA/IVB/unknown)	10/26/10/19	11/21/16/17	0.905	3/9/11/18/24	6/14/10/18/17	0.305
HCC (recurrence/naive)	57/8	54/11	0.460			
Total bilirubin (mg/dL)	0.8 (0.6–1.0)	0.8 (0.7–1.0)	0.445	0.9 (0.7–1.3)	1.1 (0.7–1.9)	0.358
Albumin (g/dL)	3.8 (3.4–4.1)	3.6 (3.4–3.9)	0.125	**3.6 (3.2–3.8)**	**3.4 (2.9–3.7)**	**0.035**
Prothrombin time-international normalized ratio	1.05 (0.97–1.15)	1.05 (0.98–1.12)	0.817	1.03 (0.96–1.12)	1.02 (0.98–1.20)	0.799
Platelet count (× 10^4^/μL)	14.1 (10.0–17.7)	15.0 (10.8–18.9)	0.568	13.2 (8.5–17.5)	13.1 (10.2–18.8)	0.351
Choline-esterase (U/L) **	**216 (171–264)**	**173 (138–244)**	**0.024**	**190 (145–257)**	**150 (107–197)**	**0.001**
Total cholesterol (mg/dL)**	170 (147–189)	162 (149–177)	0.380	168 (130–204)	158 (121–204)	0.468
Triglyceride (mg/dL)**	91 (70–129)	104 (77–135)	0.219	98 (62–147)	82 (68–108)	0.646
LDL-L (mg/dL)**	85 (64–114)	93 (70–115)	0.478	97 (71–128)	92 (63–115)	0.446
Hemoglobin A1c (%)**	5.7 (5.5–6.5)	5.9 (5.5–6.5)	0.761	5.6 (5.3–6.1)	5.3 (5.1–6.1)	0.322
Ammonia (μg/dL)**	**46 (34–55)**	**53 (41–66)**	**0.040**	45 (30–52)	47 (34–66)	0.164
AFP (ng/dL)	31.8 (5.8–415)	97.2 (8.5–2156)	0.181	64.1 (5.1–1574)	59.8 (6.5–1875)	0.571
PIVKA-II (mAU/mL)	424 (39–1719)	663 (80.3–2136)	0.368	1825 (296–17,882)	2251 (210–16,902)	0.837
Initial dose of LEN (4/8/12 mg)	**2/35/28**	**6/41/18**	**0.037**			
Initial dose down (yes/no)	15/50	22/43	0.176			
Dose down during administration (yes/no/unknown)				**37/25/3**	**26/38/1**	**0.033**
Administration period of LEN (months)				**9.4 (4.6–13.8)**	**6.0 (2.7–9.4)**	**0.015**
Discontinued LEN (yes/no)				48/17	49/16	0.843
Reason for discontinuing LEN (adverse event/PD/other) (*n* = 97)				16/26/6	23/18/8	0.175
Receiving other treatments after discontinuing LEN (yes/no) (*n* = 97)				**37/11**	**22/27**	**0.001**
The 1st mRECIST assessment (CR/PR/SD/PD/unknown)	0/25/16/20/4	0/20/22/22/1	0.446			
Death (yes/no)				22/43	30/35	0.154
PMI (cm^2^/m^2^)	5.58 (4.48–7.22)	5.66 (4.79–6.61)	0.773	**5.91 (4.38–7.03)**	**4.26 (3.59–5.32)**	** < 0.001**
ΔPMI/m (cm^2^/m^2^)				–0.01 (–0.13–0.3)	0.19 (0.13–0.45)	< 0.001

Significant values are in [bold].

Values are presented as median (interquartile range). *LEN* lenvatinib, *BMI* body mass index, *ALBI* albumin-bilirubin, *mALBI* modified albumin-bilirubin, *TNM* tumor node metastasis, *HCC* hepatocellular carcinoma, *LCL-C* low density lipoprotein cholesterol, *AFP* alpha fetoprotein, *PIVKA-II* protein induced by Vitamin K absence or antagonists-II, *PD* progressive disease, *mRECIST* Modified Response Evaluation Criteria in Solid Tumors, *CR* complete response, *PR* partial response, *SD* stable disease, *PMI* psoas muscle index, *ΔPMI/m* change in PMI per month.

Severe atrophy group: ΔPMI/m rate ≥ 1%, Mild atrophy group: ΔPMI/m rate < 1%.

(ΔPMI/m rate: rate of change in PMI per month during administration of LEN).

**Calculated using the available data.

During the LEN administration, the LEN dose was reduced in fewer patients in the severe atrophy group compared with the mild atrophy group (*P* = 0.033). Fewer patients in the severe atrophy group received other treatments after discontinuing LEN compared with those in the mild atrophy group (*P* = 0.001). There was no significant difference in other HCC-related factors such as TNM stage and tumor markers between the groups at the start or end of administration of LEN.

### Adverse events

An adverse event (AEs), regardless of grade, was experienced by 123 patients (94.6%), and severe AEs (grade 3 or worse) occurred in 37 patients (28.5%). There was no significant difference in any AEs, regardless of grade, between the low and normal PMI groups (Table S1). There was a significant difference in severe hypertension between the severe and mild atrophy groups (Table S2). There was no significant difference in the rate of severe AEs between the low and normal PMI groups (28.6% vs. 28.3%, *P* = 0.975) or between the severe and mild muscle atrophy groups (23.1% vs. 33.8%, *P* = 0.14). There was no significant difference in BMI between patients with and without severe AEs (*P* = 0.73).

There was no difference in PMI-Pre or ΔPMI/m rate between patients who discontinued due to adverse events or progressive disease (*P* = 0.367 and *P* = 0.15, respectively).

## Discussion

In this study, we assessed change in SMV in patients with HCC who were treated with LEN. We found that SMV decreased during the administration period and that change in SMV mass during the administration period was significantly associated with poor prognosis in these patients.

Sarcopenia is associated with poor prognosis in patients with cirrhosis or HCC, independent of liver function reserve^9–14^. Patients with HCC receive various treatments, along with assessment of both TNM stage and liver function reserve. In terms of treatment of HCC, sarcopenia is associated with a negative impact in HCC patients who undergo curative treatments such as hepatectomy and radiofrequency ablation^18^. Most of these previous studies assessed SMV prior to initiation of treatment. Other non-curative treatment is often continued or repeated until response failure or intolerance to treatment occurs. The results of the present study suggest that because SMV can change during non-curative treatment (Fig. 1), the impact of SMV may be less at the start of non-curative treatment compared with its impact during curative treatment. Several studies have reported no significant association between sorafenib and hepatic intra-arterial therapy with SMV loss at baseline or OS, but identified change in SMV during these treatments as a significant prognostic factor for OS^15,16^. Furthermore, Roch et al. reported that the dynamic change in SMI (a decrease of 5% or more of the L3 SMI) during the first eight weeks appeared as a stronger prognostic factor than sarcopenia at start of ICI^19^. In contrast, Imai et al. reported an association of SMV loss with poor prognosis, both at baseline and during treatment^12^. These findings imply that change in SMV during treatment may be a useful predictor of prognosis in patients with HCC receiving non-curative treatment, including LEN.

Several studies have identified change in SMV during treatment as an impact factor in HCC patients with treated with MTAs^12,15^. However, these reports did not consider the impact of long-term change in SMV. In the present study, the median administration period of LEN was 7.7 months, and the impact of long-term change in SMV over at least 6 months was investigated in more than half of the enrolled patients.

Tsung et al. reported that pre-sarcopenia was associated with worse PPS and patients who sustained normal muscle mass from the beginning of sorafenib treatment to treatment failure had better PPS than those who developed pre-sarcopenia^20^. In this study as well, patients in the severe atrophy group had significantly worse PPS than those in the mild atrophy group, which was similar to the previous reported results. Prevention of SMV loss was associated to the better post progression outcome.

In patients with HCC, SMV is affected by both liver function reserve and tumor-related factors in addition to age or sex^21^. In fact, in the present study, median PMI-Pre, PMI-1st, and PMI-Post were 5.62, 5.61, and 4.94 cm^2^/m^2^, respectively, and SMV had decreased significantly between Pre and Post. Furthermore, there was no significant difference in ΔPMI/m between the low and normal PMI groups. Similarly, Uchikawa et al. reported a significant decrease in SMV during administration of MTAs, with or without muscle volume loss at baseline^22^. Roch et al. suggest that cachexia (a body-weight loss of 5% or more) might be a determinant of poor outcome of ICI^19^. In the present study, liver function reserve was worse and ChE and albumin levels were lower in the severe atrophy group than in the mild atrophy group (Table 4). These results suggest that maintaining nutritional status and hepatic reserve prevents SMV loss. Takada et al. reported that branch-chain amino acids (BCAAs) were useful for maintaining the serum albumin level, which helped to avoid early discontinuance of sorafenib therapy^23^. Another study reported that in HCC patients treated with LEN, there was significant correlation between the plasma acyl carnitine-to-free carnitine ratio and change in the Brief Fatigue Inventory score, and mentioned that LEN affected carnitine insufficiency and fatigue^24^. Accordingly, we should consider the early introduction of nutrition therapy, including BCAAs and levocarnitine, in HCC patients treated with MTAs.

In this study, non-mALBI 1 or 2a at the start of LEN was a significant risk factor for OS (Table 3). Furthermore, the median ALBI score at baseline was − 2.48 (mALBI grade 2a) in patients with mild atrophy and − 2.31 (mALBI grade 2a, but close to 2b) in those with severe atrophy (Table 4). Hiraoka et al. reported that mALBI grade 1 or 2a were better prognostic factors in LEN treatment ^25^, and similar results were obtained in this study. The above findings suggest that a decrease in hepatic reserve leads to a rapid decrease in SMV, leading to a worsening of prognosis.

In the present patients with hypertension, severe AEs (grade 3 or worse) occurred more frequently in those in the mild than those in the severe atrophy group (Table S2), despite no significant difference in any grade of hypertension between the two groups. It is possible that blood pressure control was originally worse in more of the patients in the mild atrophy group compared with the severe atrophy group. There was no significant difference in any of the other AEs that affect patients’ nutritional status such as diarrhea, anorexia, or body weight loss between either of the low and normal PMI groups or the severe and mild atrophy groups. Uojima et al. have reported low SMV as a risk factor associated with severe AEs in patients treated with LEN, and that SMV was more important than body weight in those patients^14^. In the present study, the initial dose of LEN and dose of LEN during administration were reduced as appropriate at the discretion of each researcher, and accordingly, AEs were unlikely to occur. However, doses of LEN during administration were less reduced in patients in the severe atrophy group compared with those in the mild atrophy group. Hiraoka et al. reported that the AE of appetite loss was more frequent in patients with lower body mass index (BMI) when a set dose was used and proposed the need for dose adjustment according to the conditions^26^. Although the cause of our result could not be determined because the decision to reduce the dose was left to each researcher, it is possible that more appropriate volume adjustment was performed according to the patient's condition in the mild atrophy group than the severe atrophy group. In the present study, lower BMI was not a risk factor for OS (Table 3) and there was no significant difference in BMI in terms of the severity of AEs. However, BMI tended to be low in the patients with severe atrophy than in those with mild atrophy (Table 4). Furthermore, the Pre values of ChE were lower in the severe atrophy group than in the mild atrophy group. These results suggest that patients with low BMI or poor nutritional status need to adjust their LEN dose more carefully.

The present study had several limitations in addition to its retrospective design and small sample size. First, as markers of muscular strength such as hand grip strength and walking speed were not evaluated, a diagnosis of sarcopenia was impossible according to the JSH guidelines. Second, we measured PMI by manual tracing on the CT images, which could have introduced errors due to inconsistency among the researchers. Third, we evaluated nutritional status only with respect to albumin, sugar metabolism, lipid metabolism and ChE, and BCAAs supplementation was not examined. Sano et al. have reported a significant association of BCAAs levels with sarcopenia, and a significant correlation of Δleucine with ΔSMI (R = 0.256, *P* < 0.001)^27^. Okubo et al. have reported that sarcopenia was diagnosed significantly more frequently in patients with vitamin D deficiency compared with those without vitamin D deficiency^28^. In addition, we did not evaluate liver fibrosis by hyaluronic acid, Mac-2 binding protein glycosylated isomer, or elastography (e.g., transient elastography, magnetic resonance). To prevent sarcopenia and thus improve prognosis in HCC patients treated with LEN, prospective large-scale and interventional studies are needed to overcome the above-mentioned limitations.

In conclusion, progressive loss of SMV was associated with OS in patients with HCC during administration of LEN. Prevention of SMV loss, including nutrition therapy, is essential for improving prognosis in HCC patients treated with LEN.

## Methods

### Patients

We retrospectively analyzed 171 patients with advanced u-HCC who were treated with LEN between April 2018 and July 2020 at one of the following hospitals in Japan: Fukushima Medical University Hospital, Iwate Medical University Hospital, Tohoku University Hospital, Yamagata University Hospital, Akita University Hospital, Hirosaki University Hospital, and National Hospital Organization Sendai Medical Center. Patients who had received other treatments such as surgery, local therapy, hepatic intra-arterial therapy, radiation therapy, and chemotherapy including sorafenib before the start of LEN administration were included in the study. Excluded were 39 patients in whom a large amount of data were missing at the start of LEN, or in whom SMI could not be measured because magnetic resonance imaging (MRI) was performed instead of CT. Propensity score matching was performed, and 130 patients were enrolled in the study.

All enrolled patients had been treated only with LEN from the start of LEN administration (Pre) to the end of LEN administration (Post). If a patient’s liver function was good enough to permit administration of other treatments (MTAs, hepatic arterial infusion chemotherapy, or radiation therapy) at Post, these treatments were added to the regimen. The study protocol conformed to the ethics guidelines of the Declaration of Helsinki. The study protocol was reviewed and opt-out consent was approved by the Ethics Committee of Fukushima Medical University (No. 2019–233). The need to obtain informed consent from the participants was waived by the Ethics Committee of Fukushima Medical University due to the retrospective nature of the study.

### Evaluation of HCC

The diagnosis of HCC was performed by CT or MRI. TNM stage was determined according to the Japanese criteria^29^. The size and number of HCCs were evaluated using the Up-to-7 criteria^30^. Therapeutic effect of LEN was assessed by CT at 1–3 months after the start of administration of LEN according to the Modified Response Evaluation Criteria in Solid Tumors (mRECIST)^31^. Afterwards, patients were followed up by CT every 3 months.

### Assessment of liver function reserve

Liver function reserve was evaluated by the Child–Pugh classification^32^, ALBI score, and mALBI grade^33,34^.

### Treatment

All patients received 4 mg/8 mg/12 mg LEN once daily based on their body weight and liver function reserve. The initial dose of LEN was reduced as appropriate, at the discretion of each researcher. When any grade 3 or worse severe AEs or any unacceptable grade 2 AEs occurred, the dose of LEN was reduced or withdrawn until symptoms subsided, according to the guidelines provided by the manufacturer. AEs were assessed according to the National Cancer Institute Common Terminology Criteria for Adverse Events, version 4.0. The administration period (months) was defined as the period between the times of the start and end of LEN administration. If any of the following criteria were met, the administration of LEN was discontinued: (i) development of any unacceptable or serious AEs, (ii) progressive disease (PD) of the tumor as defined by mRECIST, (iii) worsening liver function reserve, or (iv) request by the patient to discontinue LEN.

### Evaluation of change in skeletal muscle volume and characteristics

The JSH guidelines for sarcopenia in liver disease recommend evaluation by SMI as the total muscle volume of four limbs measured by bioelectrical impedance analysis (BIA) divided by height squared^17^. A simpler method for determining SMI is by PMI, calculated as the left–right sum of the major × minor axis of the psoas muscle at L3 on CT, divided by height squared ^16^, which is indirectly correlated with SMI obtained using BIA^17^. Thus, we used PMI in the present study. We measured PMI on CT images obtained at the start of LEN administration (PMI-Pre), at the time of the first judgement of therapeutic effect (PMI-1st), and at the end of LEN administration (PMI-Post). Patients were classified into two groups based on the cut-off values specified by JSH guidelines for sarcopenia in liver disease: low PMI group (PMI < 6.0 cm^2^/m^2^ for men and < 3.4 cm^2^/m^2^ for women) and normal PMI group (PMI ≥ 6.0 cm^2^/m^2^ for men and ≥ 3.4 cm^2^/m^2^ for women)^17^.

As an index of progressive muscle atrophy, we calculated ΔPMI/m with the following formula: (PMI-Pre – PMI-Post)/administration period^16^. The rate of decrease in PMI per month (ΔPMI/m rate; ΔPMI*100/PMI-Pre) was also calculated. Since the median ΔPMI/m rate was 0.99%, we set the cutoff value to 1%. Patients were classified into two groups (severe and mild atrophy groups) according to ΔPMI/m of ≥ 1% or < 1%, respectively.

Characteristics including age, liver function reserve, stage, laboratory findings and prognosis including OS (months), MST, and survival rate were evaluated.

### Propensity score matching

As this study was a retrospective study, several selection biases or cofounding factors existed. Propensity score matching was performed to exclude biases of confounding factors. We selected the following 4 factors: age, gender, ALBI score, and the stage of HCC, which were considered as prognostic factors for HCC patients by previous study^21^. The calculation of propensity scores determined as 0.2 times a standard deviation of all patients’ propensity scores.

### Statistical analysis

Continuous variables are expressed as the median and interquartile range (IQR). Statistical analyses were performed using the χ^2^ test, Fisher’s exact test, the Mann–Whitney U test, Kruskal–Wallis rank sum test, or Wilcoxon signed rank test, as appropriate. OS was evaluated by Kaplan–Meier analysis, using the log-rank test. Cox proportional hazard analysis was used to determine the risk factors for OS. In the case of missing values, statistical analysis was performed with the available data. All *P*-values were two-tailed, and *P*-values < 0.05 were considered statistically significant. Statistical analyses were performed using Easy R (http://www.jichi.ac.jp/saitama-sct/SaitamaHP.files/statmed.html)^35^.

## Supplementary Information

Supplementary Tables.

## Data Availability

All data generated or analyzed during this study are included in this article. Further inquiries can be directed to the corresponding author.
