# Supplementary material for: Influence of skeletal muscle volume loss during lenvatinib treatment on prognosis in unresectable hepatocellular carcinoma: a multicenter study in Tohoku, Japan
Source: Sci Rep. 2022 Apr 20;12:6479. doi: 10.1038/s41598-022-10514-3 (PMC9021276; doi:10.1038/s41598-022-10514-3)
Supplement: Supplementary file 1 — Supplementary Tables. [file 41598_2022_10514_MOESM1_ESM.pdf]

Table S1. Adverse events (AEs) in the low and normal PMI groups

	Grade 3 or worse			Any grade		
	normal PMI (n = 67)	Low PMI (n = 63)	P value	normal PMI	Low PMI	P value
Hypertension	2 (3.0%)	3 (4.8%)	0.603	24 (35.8%)	25 (39.7%)	0.783
Diarrhea	2 (3.0%)	1 (1.6%)	0.605	14 (20.9%)	13 (20.6%)	0.978
Anorexia	1 (1.5%)	4 (6.3%)	0.153	25 (37.3%)	24 (38.1%)	0.922
Fatigue	1 (1.5%)	4 (6.3%)	0.153	29 (43.3%)	19 (30.2%)	0.169
Hoarseness	0 (0%)	0 (0%)		10 (14.9%)	11 (17.5%)	0.694
Hypothyroidism	0 (0%)	0 (0%)		21 (31.3%)	20 (31.7%)	0.957
Body weight loss	0 (0%)	0 (0%)		10 (14.9%)	7 (11.1%)	0.525
Hand foot syndrome	2 (3.0%)	3 (4.8%)	0.603	27 (40.3%)	21 (33.3%)	0.521
Proteinuria	4 (6.0%)	7 (11.1%)	0.294	18 (26.9%)	15 (23.8%)	0.696
Liver damage	1 (1.5%)	1 (1.6%)	0.973	7 (10.4%)	6 (9.5%)	0.868
Hepatic encephalopathy	3 (4.5%)	1 (1.6%)	0.779	4 (5.9%)	3 (4.8%)	0.754

AEs were assessed according to the National Cancer Institute Common Terminology Criteria for Adverse Events, version 4.0.

PMI: psoas muscle index, Low PMI group: PMI <6.0 cm^2/m^2 for men and <3.4 cm^2/m^2 for women, normal PMI group: PMI ≥ 6.0 cm^2/m^2 for men and ≥ 3.4 cm^2/m^2 for women.

Table S2. Adverse events (AEs) in the severe and mild atrophy groups

	Grade 3 or worse			Any grade		
	Mild atrophy (n = 65)	Severe atrophy (n = 65)	P value	Mild atrophy	Severe atrophy	P value
Hypertension	**5 (7.7%)**	**0 (0%)**	**0.021**	20 (30.8%)	23 (35.4%)	0.616
Diarrhea	3 (4.6%)	0 (0%)	0.078	16 (24.6%)	11 (16.9%)	0.242
Anorexia	2 (3.1%)	3 (4.6%)	0.681	24 (36.9%)	25 (38.5%)	0.97
Fatigue	1 (1.5%)	4 (6.2%)	0.187	22 (33.8%)	25 (38.5%)	0.681
Hoarseness	0 (0%)	0 (0%)		11 (16.9%)	10 (15.4%)	0.754
Hypothyroidism	0 (0%)	0 (0%)		18 (27.7%)	23 (35.4%)	0.412
Body weight loss	0 (0%)	0 (0%)		10 (15.3%)	7 (10.8%)	0.398
Hand foot syndrome	2 (3.1%)	3 (4.6%)	0.681	22 (33.8%)	25 (38.5%)	0.681
Proteinuria	8 (12.3%)	3 (4.6%)	0.105	11 (16.9%)	14 (21.5%)	0.266
Liver damage	1 (1.5%)	1 (1.5%)	0.991	6 (9.2%)	7 (10.8%)	0.82
Hepatic encephalopathy	2 (3.1%)	2 (3.1%)	0.579	3 (4.6%)	4 (6.2%)	0.721

AEs were assessed according to the National Cancer Institute Common Terminology Criteria for Adverse Events, version 4.0.

PMI: psoas muscle index, Severe atrophy group: ΔPMI/m rate >1%, Mild atrophy group: ΔPMI/m rate <1%.
